# Firearm Violence Following the Implementation of California’s Gun Violence Restraining Order Law

**DOI:** 10.1001/jamanetworkopen.2022.4216

**Published:** 2022-04-05

**Authors:** Veronica A. Pear, Garen J. Wintemute, Nicholas P. Jewell, Jennifer Ahern

**Affiliations:** 1 Violence Prevention Research Program, Department of Emergency Medicine, University of California, Davis School of Medicine; 2 Division of Epidemiology, University of California, Berkeley School of Public Health; 3 Department of Medical Statistics, London School of Hygiene & Tropical Medicine, London, United Kingdom

## Abstract

**Importance:**

California’s gun violence restraining order (GVRO) law, implemented beginning in 2016, allows for people at high risk of harming themselves or others with a firearm to be temporarily disarmed and prevented from purchasing firearms for 3 weeks to 1 year; many states have recently enacted similar laws. The research to date is on older and more limited risk-warrant laws.

**Objective:**

To determine whether implementation of the California GVRO law was associated with decreased rates of firearm assault or firearm self-harm in a large metropolitan county between 2016 and 2019.

**Design, Setting, and Participants:**

This serial cross-sectional study including data from 28 counties used the synthetic control method to evaluate differences in firearm violence between San Diego County and its synthetic control following implementation of the California law from 2016 to 2019. San Diego County was used as the treated unit because it issued substantially more GVROs than any other county in California during the study period. A total of 27 California counties that issued no or very few gun violence restraining orders from 2016 to 2019 and that had stable rates of firearm violence between 2005 and 2015 were included in the control pool. Data were analyzed from February 2021 to July 2021.

**Exposures:**

Implementation of the statewide GVRO law in 2016 in San Diego County.

**Main Outcomes and Measures:**

Annual rates of fatal and nonfatal firearm assault injuries and firearm self-harm injuries per 100 000 people.

**Results:**

In the study period, there were 355 GVROs in San Diego county, and a median (IQR) total of 8 (3-20) GVROs per donor county. The mean difference between the observed rate in San Diego County and the estimated rate in the synthetic San Diego County, 2016-2019, was −0.74 firearm assaults per 100 000 (−13% difference) and 0.13 firearm self-harm injuries per 100 000 (3% difference). Results from in-space placebo tests suggested that these differences cannot be distinguished from variation due to chance (pseudo-*P* values from a 1-sided test: *P *for assault = .35, *P *for self-harm = .67).

**Conclusions and Relevance:**

To our knowledge, this study was the first to analyze the association between GVRO implementation and firearm violence in California and the first to evaluate the association between risk-based firearm removal laws and firearm assault in any state. GVROs were not associated with reduced population-level rates of firearm violence in San Diego County, but this may change as the number of orders increases over time; the association between GVROs and firearm violence at the individual level cannot be inferred from our findings and should be the subject of future studies.

## Key Points

**Question:**

Has implementation of the gun violence restraining order law, beginning in 2016, been associated with a reduction in firearm assault or firearm self-harm in San Diego County, California?

**Findings:**

In this cross-sectional study, the gun violence restraining order law was not significantly associated with a reduction in firearm violence of any kind during its first 4 years of implementation, 2016 to 2019.

**Meaning:**

These results suggest that gun violence restraining order implementation did not reduce population-level rates of firearm violence in San Diego County, but future studies should investigate whether there were individual-level benefits to those directly affected.

## Teaser

This cross-sectional study compares rates of firearm assault and firearm self-harm in a large metropolitan county following the implementation of California legislation allowing courts to restrict gun access for high-risk individuals.

## Introduction

Half of suicides and three-quarters of homicides in the US involve firearms, with firearm violence resulting in nearly 40 000 deaths and many more injuries in 2019 alone.^1^ Many acts of firearm violence—including two-thirds of public mass shootings—are preceded by explicit or implicit threats.^2^ However, in most states, individuals exhibiting a risk of harming others or themselves are legally permitted to possess and purchase firearms as long as they are not otherwise prohibited from ownership. Following a mass shooting that exposed this legal gap and its dangers, California adopted a targeted, risk-based policy for firearm violence prevention with its gun violence restraining order (GVRO) law,^3^ which went into effect on January 1, 2016.

Under this law, when an individual poses a significant danger of harming themselves or others and has or could obtain access to a firearm, law enforcement or family members (and some coworkers and school personnel as of September 2020) can petition a judge for a GVRO, which temporarily (for up to 21 days) prohibits the subject of the order—the respondent—from purchasing or possessing firearms and ammunition. Upon service of the order, the respondent has 24 hours to relinquish their firearms to a licensed firearm dealer or law enforcement agency. After 21 days, a hearing is held, at which time a judge determines whether the GVRO should be extended for 1 year (up to 5 years as of September 2020). At present, 19 states and the District of Columbia have GVRO-type laws, most often called “extreme risk protection orders.”

Despite the recent uptick in adoption, we know very little about whether these laws are working to prevent firearm violence. A small number of individual- and state-level studies using data from 2 states that have older and more limited risk-warrant laws (requiring the relinquishment of firearms but not prohibiting their purchase) found these laws to be associated with reductions in firearm suicide.^4,5,6^ No studies have evaluated their effectiveness at reducing firearm homicide (although we did identify several cases in which GVROs were issued for potential mass shootings, none of which were carried out^7^). In addition, to our knowledge, nonfatal firearm injuries have yet to be studied.

In this study, we sought to begin addressing these gaps by evaluating the association between high GVRO uptake and firearm violence death and injury in San Diego County between 2016 and 2019 (hereafter, San Diego), assessing fatal and nonfatal firearm assault and self-harm separately. In our previous description of GVRO use and respondent demographics, we found uptake of the law to be substantially higher in San Diego than any other county in the state,^8^ providing an opportunity to leverage within-state variation. Importantly, this variation does not reflect fundamental differences in need between counties, but is rather a result of San Diego having a local champion of the law—the city attorney—who was in a position to advance implementation in her jurisdiction.^9^ As many states have recently enacted similar laws, and the federal government is explicitly supporting their adoption,^10^ these findings should be of interest to policy makers and researchers nationwide.

## Methods

This study was approved by the California Health and Human Services Agency’s Committee for the Protection for Human Subjects; University of California, Berkeley’s Committee for the Protection for Human Subjects; and the University of California, Davis institutional review board. Informed consent requirements were waived because this was a secondary data analysis of largely deidentified data posing minimal risk. This report followed the Strengthening the Reporting of Observational Studies in Epidemiology (STROBE) reporting guideline for observational studies.

### Study Design and Sample

To estimate the association between GVRO implementation and firearm violence in San Diego, we used the synthetic control method, a quasi-experimental comparative case study design.^11^ The synthetic control method is suitable for population-level evaluations of interventions delivered to a single group or region, particularly when the parallel trends assumption of difference-in-differences designs cannot be met and/or there are concerns about unobserved time-varying confounding. Briefly, this approach is akin to difference-in-differences combined with matching: the post-GVRO rate of firearm violence in San Diego (the treated unit) is compared with the estimated outcome in the synthetic control unit, which is a convex combination of California control counties weighted to match the pre-GVRO (2005-2015) firearm violence trend in San Diego as closely as possible. In this way, the synthetic control estimates the counterfactual firearm violence trend in San Diego had GVROs not been used. More detailed explanations of the method, including its utility and implementation, have previously been published.^11,12,13,14^

As all of California was exposed to the GVRO law in 2016, but use of GVROs varied across counties, we only included in the donor pool (ie, the pool of potential control units) counties in California that both had stable rates of firearm violence and issued no or very few GVROs. We included 27 counties that met these criteria in the donor pool for the primary analysis (eMethods in the Supplement).

### Data and Measures

The primary outcomes were the annual county-level rates of firearm assault and firearm self-harm per 100 000 population. We aggregated individual-level firearm death data from the California Department of Public Health’s Comprehensive Death Files and nonfatal firearm injury data from the Office of Statewide Health Planning and Development’s emergency department and inpatient hospitalization discharge records. *International Classification of Diseases, Ninth Revision (ICD-9)* and *International Statistical Classification of Diseases and Related Health Problems, Tenth Revision (ICD-10)* codes were used to identify firearm violence (eTable 1 in the Supplement). These data constitute all deaths and nearly all hospital visits in the state during the study period. Records were limited to California residents, and hospitalizations resulting in deaths were discarded to avoid double-counting. County-level denominators came from annual American Community Survey (ACS) population data.^15^

To maximize exchangeability, we included county-level factors associated with firearm violence, identified a priori based on prior studies,^16,17,18,19^ in the synthetic control model. These included percentage of residents aged 15 to 24 years and 55 years and older; percentage Hispanic, non-Hispanic Black, and non-Hispanic White residents; and percentage men; nonfirearm violent crime and property crime rates per 1000 residents; handgun sales per 1000 residents; unemployment in the civilian population aged 16 years and older; and urbanicity (eMethods in the Supplement).

### Statistical Analysis

We used the synthetic control method, which assigned weights to counties in the donor pool such that the mean squared prediction error (MSPE) was minimized in the pre-GVRO period with respect to firearm violence and variables associated with firearm violence. The weighted mean of the counties was then used to estimate the firearm violence trend in the post-GVRO period in the absence of GVRO implementation. We included the pre-GVRO outcome variable for every other year in our model to help control for unmeasured confounding.^11^ In addition to the assumption that there is a weighted combination of units from the donor pool that will provide a good preintervention match to the treated unit, key assumptions of the synthetic control method are that there is no spillover between the treated unit and the untreated units in the donor pool, and there are no external shocks that would differentially affect the outcome between the treated unit and donor pool units.^13^

To determine whether the difference in firearm violence between San Diego and its synthetic control after 2016 was due to chance, we conducted “in-space” placebo tests,^14^ iteratively assigning the intervention to each control county and repeating the synthetic control procedure. Counties with a poor pre-GVRO fit, defined as having an MSPE greater than 20 times San Diego’s MSPE, were then dropped.^11,20^ The remaining counties provided the distribution of the estimator under the null. We calculated the test statistic as the mean post-GVRO difference in the rate of firearm violence in the treated unit and its synthetic control and derived the pseudo-*P* value for a 1-sided test by dividing the number of counties with a difference equal to or more negative than San Diego’s by the number of remaining placebo counties plus 1.^21^ Twenty-three counties were included in the pseudo-*P* value calculation for firearm assault and 15 counties were included in the calculation for firearm self-harm in the primary analyses. As we hypothesized that the GVRO law would be associated with a reduction in firearm violence, a 1-sided test was appropriate.

We repeated these procedures in 2 secondary analyses. First, we evaluated the county-level rate of firearm violence per 100 000 population, pooling firearm assault and self-harm at each time point. As this pooling increased the count of firearm violence events, we were able to model these outcomes biannually (every 6 months). Along with the aforementioned factors, we included the pre-GVRO outcome variable for every other biannual period in these models.

Second, we evaluated outcomes by race and ethnicity among groups with enough GVROs to support the possibility of a population-level association. Specifically, we evaluated firearm assault among Black and Hispanic individuals (pooled), firearm assault among non-Hispanic White individuals, and firearm self-harm among non-Hispanic White individuals. Models were otherwise specified identically to those in the primary analyses.

We also conducted 3 sensitivity analyses to test the robustness of our findings. First, we repeated the primary analyses using a more restrictive donor pool, which, as before, included only counties with a stable rate of firearm violence, but now excluded counties that ever had 10 or more GVRO respondents in a year, given the previously estimated number needed to treat of 10 to 20 for firearm suicide.^4,5^ This gave us a donor pool of 20 counties.

Second, we repeated the primary analyses using 2018 as the year of implementation because it was not until then that GVROs were being used in large numbers in San Diego (which had fewer than 10 respondents in 2016 and 2017, about 75 in 2018, and over 250 respondents in 2019).^8^ For missing data in 2016 and 2017 (eg, handgun sales rate, crime rates, and percentage unemployed), values were carried forward from 2015.

Finally, we tested the primary associations of interest using controlled interrupted time series analyses, with synthetic San Diego serving as the control group (eMethods in the Supplement). This allowed for the quantification of the differences in both level and slope changes post–GVRO implementation. Analyses were performed with R version 4.0.2 (R Project for Statistical Computing).

## Results

From 2016 to 2019, there were 355 GVRO respondents in San Diego and a median (IQR) total of 8 (3-20) GVROs per donor county. Two hundred ten respondents were non-Hispanic White, 114 were Black or Hispanic, and 31 identified their race as not White, Black, or Hispanic. During the same period, there were 648 injuries and deaths from firearm assault and 696 from firearm self-harm in San Diego. Four hundred fifty-three firearm assault injuries (69.9%) were among Black and Hispanic residents and 124 (19.1%) were among non-Hispanic White residents. Five hundred twenty-five self-harm injuries (75.4%) were among non-Hispanic White residents.

### Annual Firearm Assault

**Figure 1.**
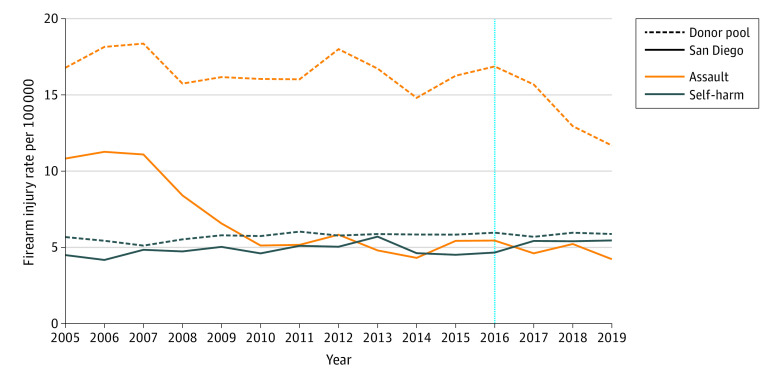
Mean Annual Rate of Firearm Assault and Self-harm in Donor Pool Counties and in San Diego The vertical dotted line denotes the start of the gun violence restraining order implementation period.

San Diego had lower rates of firearm assault throughout the study period compared with the mean rates in the donor pool (Figure 1). Firearm assault rates dropped precipitously in San Diego between 2007 and 2010, decreasing by 6.0 injuries per 100 000 population, while rates in the donor pool had a similar decline of 5.2 injuries per 100 000 between 2016 and 2019.

Seven donor pool counties were assigned nonzero weights in the firearm assault analysis (eTable 2 in the Supplement). The covariate balance between San Diego and its synthetic control (Table 1) showed that variables were well-matched, with the exception of the percentage of non-Hispanic White (15% difference) and Hispanic residents (7% difference). However, the pre-GVRO synthetic control model fit was reasonable (Table 2).

**Table 1.** Mean Annual Estimated Values Pre-GVRO Implementation by Model

Variable	Residents, %			
	San Diego	Synthetic control		Donor pool mean
		Assault	Self-harm	
Age				
15-24 y	15.67	14.86	15.05	14.89
≥55 y	22.17	27.76	22.98	23.47
Race and ethnicity				
Non-Hispanic White	48.92	64.09	46.97	48.67
Non-Hispanic Black	4.83	2.62	4.61	4.68
Hispanic	31.74	24.67	34.56	33.36
Men	50.21	49.82	49.82	49.97
Unemployed	8.29	8.48	8.83	10.35
Urbanicity^a^	1.00	1.69	1.47	1.85
Property crime rate^b^	24.51	21.38	25.25	30.44
Nonfirearm violent crime rate^b^	3.12	3.08	2.60	3.49
Handgun sales rate^b^	5.43	5.18	5.76	6.52

^a^ Urbanicity scale per the 2013 Rural-Urban Continuum Code, as assigned by the US Department of Agriculture. Values range from 1 (most urban) to 9 (most rural).

^b^ Annual rates are per 1000 residents.

**Table 2.** Synthetic Control Results, Primary Analyses

Results	Annual rate per 100 000 residents	
	Firearm assault	Firearm self-harm
San Diego	4.87	5.23
Synthetic San Diego	5.61	5.10
Rate difference	−0.74	0.13
% difference	−13	3
Pseudo *P* value^a^	.35	.67
Model fit (MSPE pre-GVRO)	0.66	0.03

Abbreviations: GVRO, gun violence restraining order; MSPE, mean squared prediction error.

^a^ Twenty-three counties were included in the pseudo-*P* value calculation for firearm assault and 15 counties were included in the calculation for firearm self-harm. Calculations for pseudo-*P* value are presented in the Methods section.

**Figure 2.**
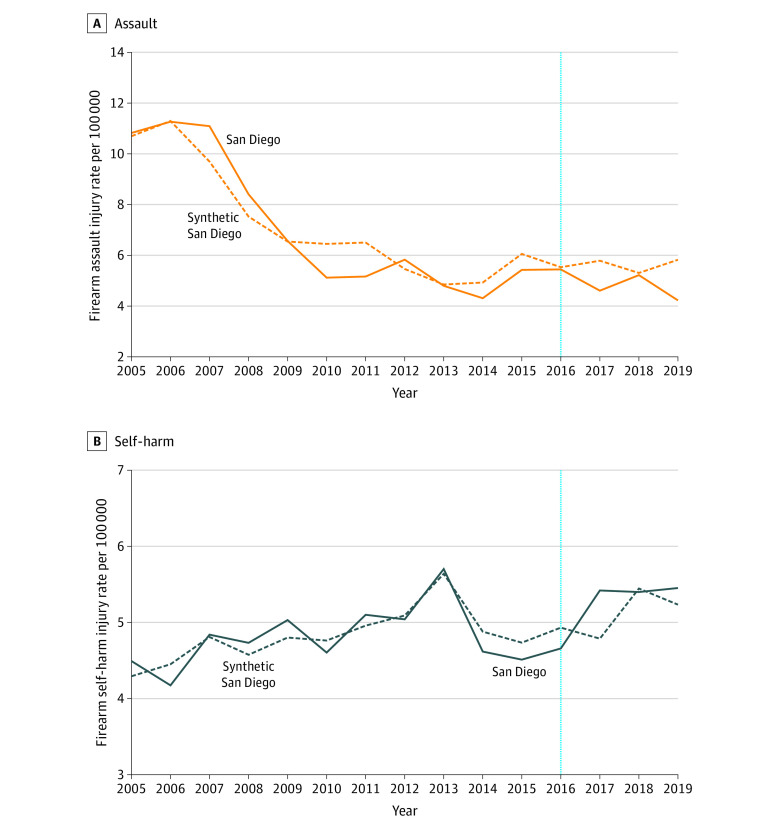
Annual Firearm Assault and Self-harm Rates in San Diego and Synthetic San Diego The vertical dotted line indicates implementation of the gun violence restraining order law.

**Figure 3.**
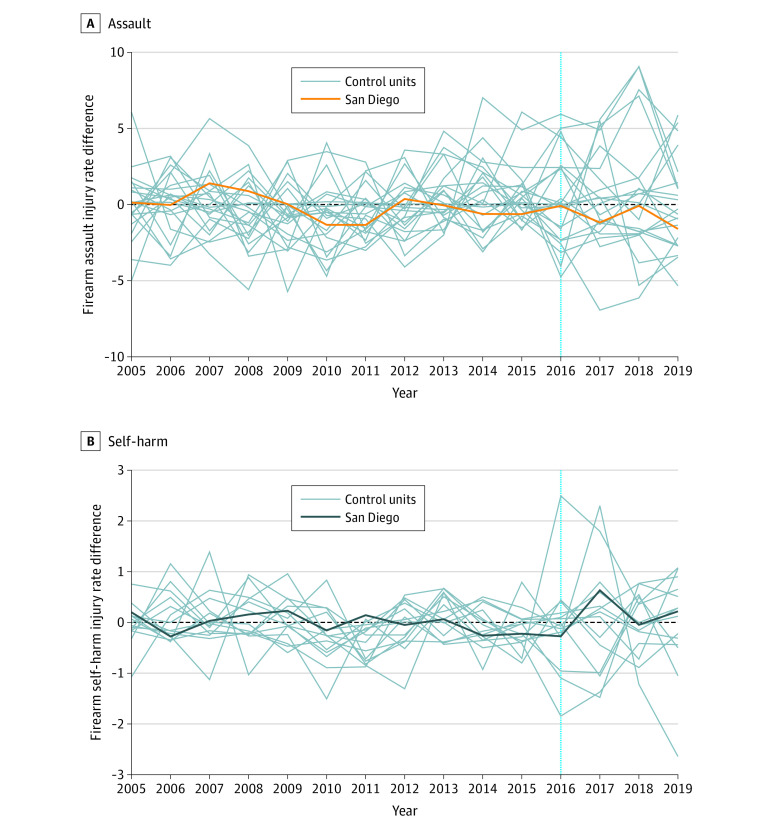
Placebo Test Results for the Association Between Gun Violence Restraining Order Implementation and Annual Firearm Assault and Self-harm Rates in San Diego Each line displays the difference between the observed and estimated rate of firearm injury. The vertical dotted line indicates the implementation of the gun violence restraining order law.

The firearm assault trend in San Diego appeared to be well-matched prior to GVRO implementation by synthetic San Diego (Figure 2A). Postimplementation, the mean firearm assault rate in San Diego (4.87 per 100 000 population) was 13% lower than the synthetic control rate (5.61 per 100 000 population) (Table 2). However, placebo test results suggested this difference was consistent with the null hypothesis of GVROs not being associated with a reduction in firearm assault in San Diego (Table 2, Figure 3A).

### Annual Firearm Self-harm

San Diego's rates of firearm self-harm injuries were similar to the mean donor pool rates—around 5 per 100 000 population—and both were stable across the study period (Figure 1). The firearm self-harm synthetic control unit was composed of 8 counties assigned nonzero weights (eTable 2 in the Supplement). The covariate balance and model fit were excellent (Table 1, Table 2).

In the synthetic control analysis firearm self-harm was 3% higher in San Diego (5.23 per 100 000) than synthetic San Diego (5.10 per 100 000) after GVRO implementation (Table 2, Figure 2B). The placebo tests suggested this result was consistent with the null hypothesis of GVROs not being associated with a reduction in firearm self-harm in San Diego (Table 2, Figure 3B).

### Secondary Analyses

As in the primary analyses, no rate differences could be distinguished from chance variation in secondary analyses (eTable 3 in the Supplement). The pre-GVRO MSPE generally suggested a good fit, with the exception of the firearm assault model for Black and Hispanic residents. As expected, the post-GVRO difference in the biannual rate of overall firearm violence fell between the outcome-specific estimates, with San Diego’s rate 1% lower than synthetic San Diego. The race and ethnicity specific models yielded larger differences than the primary models, but were not statistically significant. The percentage difference for the firearm assault models were very similar: −28% for Black and Hispanic residents and −24% for White residents. The firearm self-harm rate difference among White residents was −5%.

### Sensitivity Analyses

In the sensitivity analysis using a more restrictive donor pool, model fit was worse for firearm assault than in the models using the primary donor pool but remained the same for firearm self-harm (eTable 4 in the Supplement). Differences in the outcome were more extreme for both models, but these differences remained indistinguishable from chance variation.

In the sensitivity analysis using 2018 as the year of implementation, model fit was slightly better than in models using 2016 for firearm assault but slightly worse for firearm suicide (eTable 5 in the Supplement). Neither model suggested GVROs were associated with a reduction in firearm violence.

Finally, results from the controlled interrupted time series analyses were consistent with the findings of the primary analyses (eTable 6 in the Supplement). They did not indicate significant differences in the level or slope of firearm assault or self-harm following implementation of the GVRO law in San Diego compared with its synthetic control.

## Discussion

To our knowledge, this is the first study to evaluate the association between risk-based firearm removal laws and fatal and nonfatal firearm injuries, including assaults. Using the synthetic control method to compare post-GVRO implementation rates of firearm violence in San Diego with estimated rates in the absence of implementation, we found no evidence that GVRO implementation was associated with decreased firearm assault or firearm self-harm at the population level in San Diego. These findings were consistent with secondary analyses and robust to alternative model specifications. Our results could reflect a true absence of association or limitations of our study; further research is needed to determine which of these is the case.

The findings of no association between GVRO implementation and either firearm assault or overall firearm violence may be partially explained by access to firearms through the underground market. It is possible that respondents in danger of harming others were more likely to be able to access firearms unlawfully and acquire illicit firearms after dispossession via a GVRO. For example, most prisoners who possessed a firearm at the time of their offense obtained their firearms from the underground market, theft, or a friend,^22^ methods that might be unaffected by a GVRO.

However, it is worth noting that the firearm assault models consistently yielded the largest estimated rate reductions following GVRO implementation. With additional power via more years of data, greater implementation, and/or a larger donor pool (perhaps looking outside of California), these differences may be distinguishable from chance.

Our self-harm findings were contrary to 2 previous state-level studies that found GVRO-type laws to be associated with reductions in firearm suicide. Kivisto and Phalen^6^ found a significant 7.5% reduction in Indiana and a nonsignificant 1.6% reduction in Connecticut in the 10 years following enactment (and Connecticut’s law was associated with a significant 16% reduction in sensitivity analyses). Using these states along with California and Washington, Saadi and colleagues^23^ found these laws to be significantly associated with a roughly 2.5% reduction in firearm suicide among persons aged 55 years and older. However, California and Washington only contributed 1 exposed year each, so these findings may not accurately reflect associations for these states.

There are several possible reasons why our findings of no association for suicide diverged from those of previous studies. Our study took place in California, which has more comprehensive firearm legislation than Indiana and Connecticut.^24^ This could result in other mechanisms preventing high-risk individuals from possessing firearms in California, lessening the need for GVROs compared with other states. This possibility is supported somewhat by the relatively slow uptake in California compared with states having fewer firearm regulations, such as Florida, which experienced rapid uptake of extreme-risk protection orders following the law’s enactment.^25^

Second, firearm suicide rates are substantially lower in California than Indiana—4.02 per 100 000 population vs 8.47 per 100 000 population, respectively, in 2019.^1^ Moreover, the proportion of suicides completed with a firearm is much larger in Indiana (59%) than California (36%).^1^ This suggests that the need for GVROs for suicide prevention may be higher in Indiana, as the underlying at-risk population seems to be larger (relative to the state population). Additionally, Indiana has a greater proportion of older White men (who are at highest risk^17,26^) and higher rates of firearm ownership, which is strongly associated with firearm suicide.^27,28^

Finally, a population-level association may simply be undetectable with such a rare exposure, although GVROs are supposed to be highly targeted to those at greatest risk and even small changes in a rare outcome can affect its rate. The association may also be diluted by the presence of firearm violence events that were not amenable to a GVRO because, for example, the shooter did not exhibit warning signs. Perhaps when more GVROs have been issued, there will be a detectable association. In the meantime, there is an urgent need for individual-level effectiveness studies, as there may be strong individual-level benefits that are not being detected at the population level.

It is also, of course, possible that we identified a true absence of effect. Perhaps GVROs were not being implemented in the right cases or all firearms were not being routinely recovered. Further research on how these orders are being implemented in practice—and how they can be more accurately targeted to those at highest risk—is warranted.

### Limitations

This study was subject to limitations. Some counties in the donor pool were not completely unexposed to GVRO implementation. We included counties only if the amount of exposure could not plausibly affect firearm violence rates. We believe the benefits of using within-state controls, which allowed us to include nonfatal injuries and to avoid between-state confounding, outweighed the bias that may stem from including control counties with a small amount of exposure. Furthermore, there could have been spillover between counties using GVROs (including but not limited to San Diego) and adjacent counties not using GVROs. This could happen, for example, if a respondent in San Diego County was prevented from harming someone in adjacent Orange County. Given the small number of GVROs used outside of San Diego and the fact that most interpersonal violence occurs between acquaintances who are physical proximate to one another, we believe the amount of spillover would be undetectable at the county level. Moreover, to the degree that control counties were exposed, our results should be biased toward the null.

It is possible that the synthetic control did not provide a good estimation of the counterfactual. This could happen if, for example, there were unmeasured differences between counties in the donor pool and San Diego that compromised comparability. Including preimplementation outcome data was intended to adjust for unobserved factors, assuming that similar trends indicate similar values with respect to variables that contribute to those trends.^11^ As our synthetic controls fit the preintervention trends reasonably well, there was not likely to be a large degree of unmeasured confounding.^21^ Residual confounding could stem from policy changes that affect firearm violence differently between San Diego and counties in the donor pool, but we know of no such changes.

Generalizability of our findings is uncertain. Since San Diego implemented far more GVROs than any other county in the state, it is likely that these null results will generalize to a statewide analysis of California. However, this might not be the case if GVROs are systematically being used in different circumstances outside of San Diego. Differences between states, such as the existence of other firearm laws, make it difficult to know whether results will generalize beyond California. However, in states with a higher demand for this type of intervention (due to fewer alternative mechanisms preventing high-risk individuals from possessing firearms), associations may be stronger.

## Conclusions

This study was the first to our knowledge to analyze the association between GVRO implementation and fatal and nonfatal firearm violence in California and to evaluate the association between risk-based firearm removal laws and firearm assault in any state. We did not find evidence for a county-level reduction in firearm assault or firearm self-harm following implementation of the law in San Diego. Given the relatively small number of GVROs issued during the study period and our small donor pool, these results should be taken as preliminary. They do not preclude the possibility of an individual-level benefit to respondents, and previous research indicates that such a benefit exists for those in danger of self-harm.^4,5^ Future research should prioritize studies of longer-term effects—which may be more pronounced as additional petitioner types are permitted and uptake increases—and studies at the individual level. Despite our null findings, the state of the evidence overall supports GVROs and related legislation as tools that may be useful in preventing firearm injury and death.

## Notes

Supplement. **eMethods.** Selecting Counties for the Donor Pool, County-Level Variable Details, and Controlled Interrupted Time Series **eTable 1.** *ICD-9* and *ICD-10* Codes Used to Identify Firearm Violence **eTable 2.** Donor Pool Weights by Model **eTable 3.** Synthetic Control Results, Secondary Analyses **eTable 4.** Synthetic Control Results, Sensitivity Analysis: More Restrictive Donor Pool (n=20) **eTable 5.** Synthetic Control Results, Sensitivity Analysis: 2018 Intervention **eTable 6.** Controlled Interrupted Time Series Results, Sensitivity Analysis
eReferences.
