# Supplementary material for: Firearm Violence Following the Implementation of California’s Gun Violence Restraining Order Law
Source: JAMA Netw Open. 2022 Apr 5;5(4):e224216. doi: 10.1001/jamanetworkopen.2022.4216 (PMC8984773; doi:10.1001/jamanetworkopen.2022.4216)
Supplement: Supplement. — eMethods. Selecting Counties for the Donor Pool, County-Level Variable Details, and Controlled Interrupted Time Series eTable 1. ICD-9 and ICD-10 Codes Used to Identify Firearm Violence eTable 2. Donor Pool Weights by Model eTable 3. Synthetic Control Results, Secondary Analyses eTable 4. Synthetic Control Results, Sensitivity Analysis: More Restrictive Donor Pool (n=20) eTable 5. Synthetic Control Results, Sensitivity Analysis: 2018 Intervention eTable 6. Controlled Interrupted Time Series Results, Sensitivity Analysis eReferences. [file jamanetwopen-e224216-s001.pdf]

Supplemental Online Content

Pear VA, Wintemute GJ, Jewell NP, Ahern J. Firearm violence following the implementation of California's gun violence restraining order law. *JAMA Netw Open.* 2022;5(4):e224216. doi:10.1001/jamanetworkopen.2022.4216

eMethods. Selecting Counties for the Donor Pool, County-Level Variable Details, and Controlled Interrupted Time Series

eTable 1. *ICD-9* and *ICD-10* Codes Used to Identify Firearm Violence

eTable 2. Donor Pool Weights by Model

eTable 3. Synthetic Control Results, Secondary Analyses

eTable 4. Synthetic Control Results, Sensitivity Analysis: More Restrictive Donor Pool (n=20)

eTable 5. Synthetic Control Results, Sensitivity Analysis: 2018 Intervention

eTable 6. Controlled Interrupted Time Series Results, Sensitivity Analysis

eReferences.

This supplemental material has been provided by the authors to give readers additional information about their work.

© 2022 Pear VA et al. *JAMA Network Open.*

eMethods

Selecting Counties for the Donor Pool

Following CDC guidelines for unstable rates,[1] we excluded 26 counties from the donor pool for having, on average, fewer than 20 firearm violence injuries per year in the pre-GVRO period. To ensure we did not include exposed counties in the donor pool, we removed an additional 4 counties with a ratio of GVROs to expected firearm violence injuries > 0.1 (calculated as: total GVRO respondents 2016-2019 / [mean annual firearm violence injuries*4]). We determined the number of GVRO respondents per county using California Restraining and Protective Order System data, maintained by the California Department of Justice (CA DOJ). The 0.1 cutoff was guided by Swanson and colleagues' finding that 1 firearm suicide was prevented for every 10-20 cases of firearm removal pursuant to a risk-warrant in Connecticut and Indiana.[2,3] Based on this figure, if 10% of the population that went on to harm themselves with a firearm was first served a GVRO, we would expect a 1% reduction in firearm self-harm. We are assuming any lesser measure of association would be undetectable at a population level, such that these counties can be considered unexposed. This left us with 27 control counties in the primary analysis.

County-Level Predictor Details

County demographic variables came from annual American Community Survey (ACS) population data.[4] Crime rates were estimated with annual publicly available Crimes & Clearances data from CA DOJ[5] and firearm sales were measured with CA DOJ's Dealer Record of Sales data, which contain records of nearly all legal handgun transfers in California. We were missing the last 3 months of DROS data in 2015, so we used the last 3 months of 2014 in its place. Crime and firearm sales were calculated per 1,000 residents. Unemployment data was measured with the ACS 5-year estimates for 2005-2009 and 2010-2014.[6] Urbanicity was measured with the 2013 Rural-Urban Continuum Codes created by the US Department of Agriculture.[7]

In the secondary analysis evaluating biannual overall firearm violence, denominators and predictors were changed to 6-month estimates to the extent possible. County-level biannual population estimates were created by linearly interpolating monthly populations (overall and for subgroups by age, sex, and race/ethnicity) between annual American Community Survey (ACS) estimates[4] and averaging the first and last 6 months of each year. ACS data for 2020 were not available, so interpolation could not be done for 2019; we therefore used the annual estimate for both biannual periods. These biannual population estimates were used as denominators and to characterize the demographic variables of interests.

Controlled Interrupted Time Series

As an additional sensitivity analysis and to test for a change in slope after GVROs were implemented, we evaluated changes in firearm assault and self-harm in San Diego relative to its synthetic control with controlled interrupted time series analyses. These took the following form:

$$Y_t = \beta_0 + \beta_1 T + \beta_2 X_t + \beta_3 T X_t + \beta_4 G + \beta_5 G T + \beta_6 G X_t + \beta_7 G X_t T$$

© 2022 Pear VA et al. *JAMA Network Open.*

Y_t is the firearm assault or self-harm rate at time t; T is a linear time trend; X is a dummy variable for the intervention, pre- or post-GVRO implementation; and G is a dummy variable for the treated (San Diego) and control group (synthetic San Diego). The coefficients of interest are β_6 and β_7.The former provides the estimated difference in the level change post-GVROs between San Diego and synthetic San Diego, and the latter provides the estimate difference in the change in slop between the two groups after the GVRO law went into effect. We used Newey-West confidence intervals to account for autocorrelation.[8]

© 2022 Pear VA et al. *JAMA Network Open.*

eTable 1. *ICD-9* and *ICD-10* Codes Used to Identify Firearm Violence[a]

	**Injury Data**		**Mortality Data**
**Description**	**ICD-9 Code (1/2005-9/2015)**	**ICD-10 Code (10/2015-12/2019)**	**ICD-10 Code**
Homicide & assault by:			
Handgun	E965.0	X93	X93
Shotgun	E965.1	X94	X94
Hunting rifle	E965.2	X94	X94
Military firearms	E965.3	X94	X94
Other and unspecified firearm	E965.4	X95.8, X95.9	X95
Suicide & self-inflicted injury by:			
Handgun	E955.0	X72	X72
Shotgun	E955.1	X73	X73
Hunting rifle	E955.2	X73	X73
Military firearms	E955.3	X73	X73
Other and unspecified firearm	E955.4	X74	X74

a. ICD = International Classification of Diseases

© 2022 Pear VA et al. *JAMA Network Open.*

eTable 2. Donor Pool Weights by Model

	**Model Outcome**	
**Donor County**	**Annual Firearm Assault**	**Annual Firearm Self-Harm**
El Dorado	0.163	0
Los Angeles	0.162	0
Placer	0.001	0.061
San Francisco	0.008	0
San Luis Obispo	0.259	0.100
Shasta	0.021	0
Sonoma	0.386	0
Alameda	0	0.148
Kings	0	0.026
Orange	0	0.160
Sacramento	0	0.002
San Bernardino	0	0.186
Ventura	0	0.318
Butte	0	0
Contra Costa	0	0
Fresno	0	0
Humboldt	0	0
Kern	0	0
Madera	0	0
Merced	0	0
Monterey	0	0
Riverside	0	0
San Joaquin	0	0
San Mateo	0	0
Solano	0	0
Stanislaus	0	0
Tulare	0	0

© 2022 Pear VA et al. *JAMA Network Open.*

eTable 3. Synthetic Control Results, Secondary Analyses

		Outcome[a]			
		**Biannual Firearm Violence**	**Annual Firearm Assault, Black and Hispanic**	**Annual Firearm Assault, NH White**	**Annual Firearm Self-Harm, NH White**
**San Diego**	**Rate in post-intervention period (per 100,000)**	5.05	8.85	2.05	8.67
**Synthetic San Diego**	**Rate in post-intervention period (per 100,000)**	5.11	12.22	2.70	9.08
**Rate difference**		-0.06	-3.37	-0.66	-0.41
**Percent difference**		-1%	-28%	-24%	-5%
**Pseudo P-value**[b]		12/23=0.52	6/25=0.24	5/18=0.28	5/16=0.31
**Model fit (MSPE)**		0.42	4.51	0.12	0.13

a. NH=Non-Hispanic
b. The proportion of counties (donor pool plus San Diego) with a difference between the observed rate and the synthetic control rate in the post-treatment period equal to or more negative than San Diego's. Counties with a poor pre-period fit (MSPE >20* San Diego's MSPE) were exclude from the denominator.

© 2022 Pear VA et al. *JAMA Network Open.*

eTable 4. Synthetic Control Results, Sensitivity Analysis: More Restrictive Donor Pool (n=20)

		**Outcome**	
		**Firearm Assault**	**Firearm Self-Harm**
**San Diego**	**Rate in post-intervention period (per 100,000)**	4.87	5.23
**Synthetic San Diego**	**Rate in post-intervention period (per 100,000)**	5.83	4.73
**Rate difference**		-0.96	0.51
**Percent difference**		-16%	+11%
**Pseudo P-value**[a]		7/18=0.39	6/7=0.86
**Model fit (MSPE)**		1.10	0.03

a. The proportion of counties (donor pool plus San Diego) with a difference between the observed rate and the synthetic control rate in the post-treatment period equal to or more negative than San Diego's. Counties with a poor pre-period fit (MSPE >20* San Diego's MSPE) were exclude from the denominator.

© 2022 Pear VA et al. *JAMA Network Open.*

eTable 5. Synthetic Control Results, Sensitivity Analysis: 2018 Intervention

		Outcome	
		**Firearm Assault**	**Firearm Self-Harm**
**San Diego**	**Rate in post-intervention period (per 100,000)**	4.72	5.43
**Synthetic San Diego**	**Rate in post-intervention period (per 100,000)**	5.53	5.61
**Rate difference**		-0.81	-0.19
**Percent difference**		-15%	-3%
**Pseudo P-value[a]**		11/23=0.48	8/20=0.40
**Model fit (MSPE pre-GVRO)**		0.62	0.06

a. The proportion of counties (donor pool plus San Diego) with a difference between the observed rate and the synthetic control rate in the post-treatment period equal to or more negative than San Diego's. Counties with a poor pre-period fit (MSPE >20* San Diego's MSPE) were exclude from the denominator.

© 2022 Pear VA et al. *JAMA Network Open.*

eTable 6: Controlled Interrupted Time Series Results, Sensitivity Analysis

	**Annual Firearm Assault**	**Annual Firearm Self-Harm**
Level change difference post-GVRO implementation[a]	3.32 (-1.02, 7.67)	-1.06 (-3.02, 0.90)
Slope change difference post-GVRO implementation[a]	-0.23 (-0.71, 0.25)	0.10 (-0.09, 0.29)

a. Difference is between San Diego and synthetic San Diego.

© 2022 Pear VA et al. *JAMA Network Open.*

REFERENCES

1. Annotation and suppression of unstable rates. Centers for Disease Control and Prevention, National Center for Injury Prevention and Control. 2018. Accessed December 1, 2019. www.cdc.gov/injury/wisqars/mapping_help/unstable_rates.html
2. Swanson JW, Easter MM, Alanis-Hirsch K, et al. Criminal justice and suicide outcomes with Indiana's risk-based gun seizure law. *J Am Acad Psychiatry Law.* 2019;47(2):188-197. doi:10.29158/JAAPL.003835-19
3. Swanson JW, Norko MA, Lin H, et al. Implementation and effectiveness of Connecticut's risk-based gun removal law: Does it prevent suicides? *Law Contemp Probl.* 2017;80(2):179-208.
4. ACS 2005-2019 (1-year estimates). US Census Bureau, Social Explorer. 2021. www.socialexplorer.com
5. Crimes & clearances. California Department of Justice. Accessed February 10, 2020. https://openjustice.doj.ca.gov/exploration/crime-statistics/crimes-clearances
6. ACS 2009 & 2014 (5-year estimates). US Census Bureau,Social Explorer. www.socialexplorer.com
7. Rural-urban continuum codes: Documentation. United States Department of Agriculture. 2020. Accessed July 14, 2021. www.ers.usda.gov/data-products/rural-urban-continuum-codes/documentation/
8. Bottomley C, Scott JAG, Isham V. Analysing interrupted time series with a control. *Epidemiologic Methods.* 2019;8(1). doi:doi:10.1515/em-2018-0010

© 2022 Pear VA et al. *JAMA Network Open.*
